# Impairment of Electron Transfer Chain Induced by Acute Carnosine Administration in Skeletal Muscle of Young Rats

**DOI:** 10.1155/2014/632986

**Published:** 2014-05-04

**Authors:** José Roberto Macarini, Soliany Grassi Maravai, José Henrique Cararo, Nádia Webber Dimer, Cinara Ludvig Gonçalves, Luiza Wilges Kist, Mauricio Reis Bogo, Patrícia Fernanda Schuck, Emilio Luiz Streck, Gustavo Costa Ferreira

**Affiliations:** ^1^Laboratório de Erros Inatos do Metabolismo, Programa de Pós-Graduação em Ciências da Saúde, Unidade Acadêmica de Ciências da Saúde, Universidade do Extremo Sul Catarinense, 88801-600 Criciúma, SC, Brazil; ^2^Laboratório de Bioenergética, Programa de Pós-Graduação em Ciências da Saúde, Unidade Acadêmica de Ciências da Saúde, Universidade do Extremo Sul Catarinense, 88806-000 Criciúma, SC, Brazil; ^3^Laboratório de Biologia Genômica e Molecular, Faculdade de Biociências, Pontifícia Universidade Católica do Rio Grande do Sul, 90619-900 Porto Alegre, RS, Brazil; ^4^Programa de Pós-Graduação em Medicina e Ciências da Saúde, Pontifícia Universidade Católica do Rio Grande do Sul, 90619-900 Porto Alegre, RS, Brazil; ^5^Instituto Nacional de Ciência e Tecnologia Translacional em Medicina (INCT-TM), 90035-003 Porto Alegre, RS, Brazil; ^6^Laboratório de Neuroquímica, Instituto de Biofísica Carlos Chagas Filho, Universidade Federal do Rio de Janeiro, Avenida Carlos Chagas Filho 373, Cidade Universitária, Ilha do Fundão, 21941-902 Rio de Janeiro, RJ, Brazil

## Abstract

Serum carnosinase deficiency is an inherited disorder that leads to an accumulation of carnosine in the brain tissue, cerebrospinal fluid, skeletal muscle, and other tissues of affected patients. Considering that high levels of carnosine are associated with neurological dysfunction and that the pathophysiological mechanisms involved in serum carnosinase deficiency remain poorly understood, we investigated the *in vivo* effects of carnosine on bioenergetics parameters, namely, respiratory chain complexes (I–III, II, and II-III), malate dehydrogenase, succinate dehydrogenase, and creatine kinase activities and the expression of mitochondrial-specific transcription factors (*NRF-1, PGC-1*α**, and *TFAM*) in skeletal muscle of young Wistar rats. We observed a significant decrease of complexes I–III and II activities in animals receiving carnosine acutely, as compared to control group. However, no significant alterations in respiratory chain complexes, citric acid cycle enzymes, and creatine kinase activities were found between rats receiving carnosine chronically and control group animals. As compared to control group, mRNA levels of *NRF-1, PGC-1*α**, and *TFAM* were unchanged. The present findings indicate that electron transfer through the respiratory chain is impaired in skeletal muscle of rats receiving carnosine acutely. In case these findings are confirmed by further studies and ATP depletion is also observed, impairment of bioenergetics could be considered a putative mechanism responsible for the muscle damage observed in serum carnosinase-deficient patients.

## 1. Introduction

Carnosine (*β*-alanyl-L-histidine) is an imidazole dipeptide abundant in skeletal muscle, as well as in central nervous system [1], cardiac muscle, kidney, stomach, and olfactory bulbs [2, 3] of most animals. Physiological levels of carnosine may reach up to 20 mM in muscle tissues [4], and its biological role remains unclear. Carnosine of human diet is uptaken by intestinal proton-coupled peptide transporter, namely, human H^+^/peptide cotransporter 1 (hPEPT1) [5]. This dipeptide is synthesized starting from its component amino acids histidine and *β*-alanine, by enzyme carnosine synthetase (EC 6.3.2.11). On the other hand, the clearance of carnosine is essentially due to the hydrolytic action of a metalloprotease, the carnosinase [6]. This enzyme exists in two isoforms. The secreted form, namely, serum carnosinase (EC 3.4.13.20), is found primarily in serum, as well as in brain and cerebrospinal fluid. The cytosolic form, tissue carnosinase (EC 3.4.13.3), is present in several tissues, including liver, kidney, and spleen, but not skeletal muscle [7]. Both carnosinase isoforms differ not only in distribution and molecular weight, but also in substrate specificity.

Serum carnosinase deficiency (OMIM number 212200) results in accumulation of carnosine and related compounds homocarnosine and anserine in plasma (20–30 *μ*mol/mL serum), urine [8], and tissues of affected patients. The clinical presentation includes tremor, myoclonic seizures, hypotonia, psychomotor retardation, and inability to social relationship [9, 10]. The coexistence of this rare enzymatic deficiency and a deletion in the long arm of chromosome 18 in a child was reported, suggesting the localization of serum carnosinase gene [11]. Currently, there are no reports of effective treatment for the disease. According to Willi and colleagues [11], diet restricted in meat ameliorates the clinical symptoms, but it does not fully eliminate them. Due to rarity of the disease, the mechanisms of tissue damage observed in affected subjects are still to be unraveled.

Such patients accumulate carnosine in tissues and body fluids and present a wide range of muscle alterations. In this scenario, the present work aimed to investigate the influence of acute and chronic carnosine administration on some parameters of energy homeostasis in skeletal muscle of young rats, namely, respiratory chain complexes (I–III, II, and II-III), citric acid cycle enzymes (succinate dehydrogenase and malate dehydrogenase), and creatine kinase activities, as well as the expression of transcription factors (*NRF-1*,* PGC-1*α*,* and* TFAM*) related to mitochondrial biogenesis.

## 2. Materials and Methods

### 2.1. Animals

Twenty-four male Wistar rats (250–300 g; age 30 days) obtained from the Central Animal House of Universidade do Extremo Sul Catarinense, Santa Catarina, Brazil, were caged in groups of six, provided with* ad libitum* commercial rat chow and water, and maintained on a 12 h light-dark cycle at a temperature of 23 ± 1°C. The animals were randomly divided into four groups (*n* = 6): sham (saline) and carnosine (100 mg/kg of body weight) acute and chronic groups. All studies were performed in accordance with the National Institutes of Health guidelines and EU Directive 2010/63/EU for animal experiments and with the approval of the Ethics Committee of Universidade do Extremo Sul Catarinense (Protocol number 67/2012).

### 2.2. Carnosine Administration

Male Wistar rats were divided into acute and chronic treatment groups. In acute administration, the animals received a single dose of carnosine (100 mg/kg of body weight) administered intraperitoneally. Twenty-four hours after administration, the rats were euthanized. In the chronic administration, the animals received a daily dose of the dipeptide (100 mg/kg of body weight) administered intraperitoneally for five days, and one hour after the last injection the rats were euthanized by decapitation without anesthesia, and the skeletal muscle was removed for subsequent biochemical analysis. The animals of control group were subjected to similar experimental conditions as carnosine group, but received vehicle (NaCl 0.9%) instead of carnosine.

### 2.3. Complexes I-CoQ-III Activities

NADH: oxidoreductase cytochrome* c* (complexes I–III) activity was determined according to Schapira and colleagues [14], by the determination of cytochrome *c* reduction at *λ* = 550 nm. Results are expressed as nmol·min^−1^·mg protein^−1^.

### 2.4. Complex II Activity

Succinate: 2,6-dichloroindophenol-oxidoreductase (complex II) activity was evaluated using the method described by Fischer et al. [15]. Complex II activity was measured by following the decrease in absorbance, due to the reduction of 2,6-dichloroindophenol at *λ* = 600 nm. Results are expressed as nmol·min^−1^·mg protein^−1^.

### 2.5. Complexes II-III Activities

Succinate: cytochrome *c* oxidoreductase (complexes II-III) activity was determined according to Fischer et al. [15]. Complexes II-III activities were measured by cytochrome *c* reduction, using succinate as substrate at *λ* = 550 nm. Results are expressed as nmol·min^−1^·mg protein^−1^.

### 2.6. Creatine Kinase Activity

Creatine kinase (EC 2.7.3.2) activity was measured in total homogenates using the method described by Hughes [16] with slight modifications [17]. Results are expressed as *μ*mol creatine·min^−1^·mg protein^−1^.

### 2.7. Succinate Dehydrogenase Activity

Succinate dehydrogenase (EC 1.3.99.1) activity was determined in homogenates according to Fischer et al. [15]. Results are expressed as nmol·min^−1^·mg protein^−1^.

### 2.8. Malate Dehydrogenase Activity

Malate dehydrogenase (EC 1.1.1.37) activity was evaluated according to Kitto [18] by following the reduction of NADH at wavelengths of excitation and emission of 340 and 466 nm, respectively. Results were calculated as nmol·min^−1^·mg protein^−1^.

### 2.9. Expression of Mitochondrial-Specific Transcription Factors

Transcript abundance of key factors involved in mitochondrial biogenesis was evaluated by quantitative real-time RT-PCR (RT-qPCR). Total RNA was isolated with Trizol reagent (Invitrogen, Carlsbad, CA, USA) in accordance with the manufacturer's instructions. The total RNA was quantified by spectrophotometry (A260/280 nm) and after treated with deoxyribonuclease I (Invitrogen) to eliminate genomic DNA contamination in accordance with the manufacturer's instructions. The cDNA was synthesized with ImProm-II Reverse Transcription System (Promega) from 1 *μ*g total RNA, following the manufacturer's instruction. Quantitative PCR was performed using SYBR Green I (Invitrogen) to detect double-strand cDNA synthesis. Reactions were done in a volume of 25 *μ*L using 12.5 *μ*L of diluted cDNA, containing a final concentration of 0.2x SYBR Green I (Invitrogen), 100 *μ*M dNTP, 1x PCR Buffer, 3 mM MgCl_2_, 0.25 U Platinum Taq DNA Polymerase (Invitrogen), 0.5 M of betaine (for* Gapd *[12]*, Hprt1 *[12]*, PGC-1*α*, *and* TFAM*), 2% of the reaction of DMSO (for* NRF-1* [13]), and 200 nM of each of reverse and forward primers (Table 1). The PCR cycling conditions were an initial polymerase activation step for 5 min at 95°C, 40 cycles of 15 s at 95°C for denaturation, 35 s at 60°C for annealing, and 15 s at 72°C for elongation. At the end of cycling protocol, a melting-curve analysis was included and fluorescence measured from 60 to 99°C and showed in all cases one single peak.* Gapd *and* Hprt1* were used as reference genes for normalization. Relative expression levels were determined with 7500 and 7500 Fast Real-Time PCR Systems Software v.2.0.6 (Applied Biosystems). The efficiency per sample was calculated using LinRegPCR 2012.3 Software (http://LinRegPCR.nl/). Relative mRNA expression levels were determined using the 2^−ΔΔCT^ method.

### 2.10. Protein Determination

Protein was measured by Lowry and colleagues [19] method, using bovine serum albumin as standard.

### 2.11. Statistical Analysis

Results are presented as mean ± standard error of mean (S.E.M.). Assays were performed in duplicate and the mean or median was used for statistical analysis. Data was analyzed using Student's* t*-test. Differences between groups were rated significant at* P* ≤ 0.05. All analyses were carried out in an IBM-compatible PC computer using the Statistica version 7 software. Molecular data were expressed as means ± S.E.M. and analyzed by Student's* t*-test for unpaired samples considering *P* < 0.05 as statistical significance.

## 3. Results

### 3.1. Energy Metabolism Is Impaired by Acute Carnosine Administration in Skeletal Muscle of Young Rats

**Figure 1:**
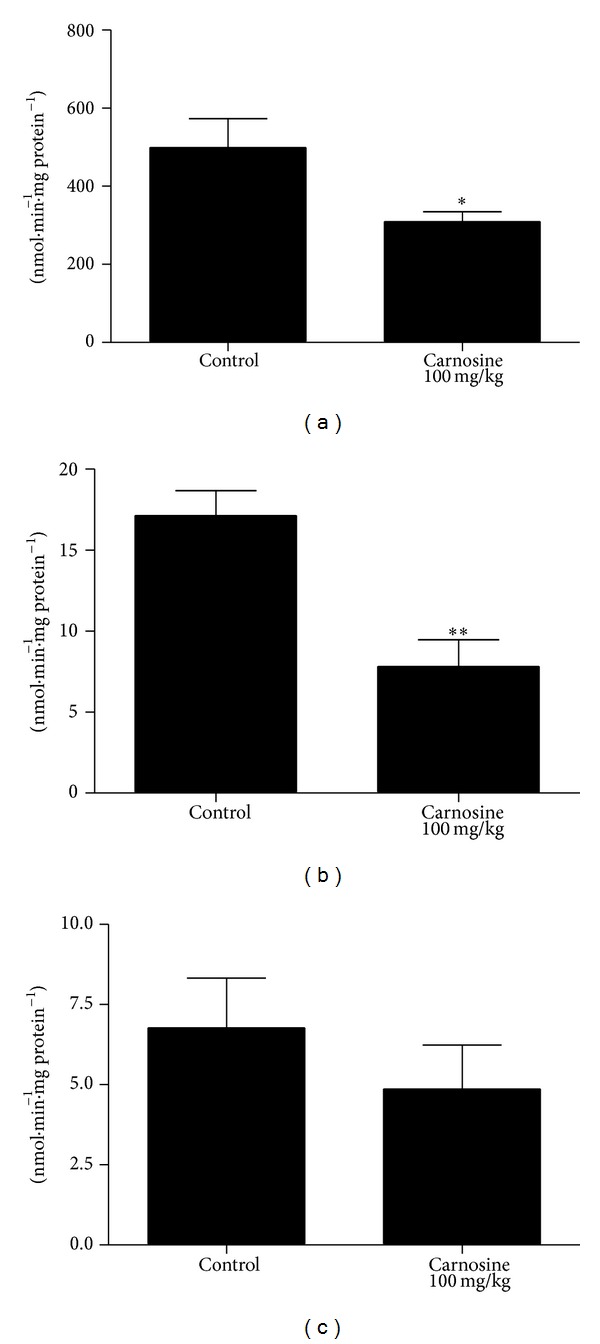
Effect of acute carnosine administration on respiratory chain complexes I–III (a), II (b), and II-III (c) activities in skeletal muscle of young rats. Data represent mean ± S.E.M. for four to six independent animals performed in duplicate and are expressed in nmol min^−1^ mg protein^−1^. *P < 0.05, **P < 0.01 compared to control group (Student's *t*-test).

**Figure 2:**
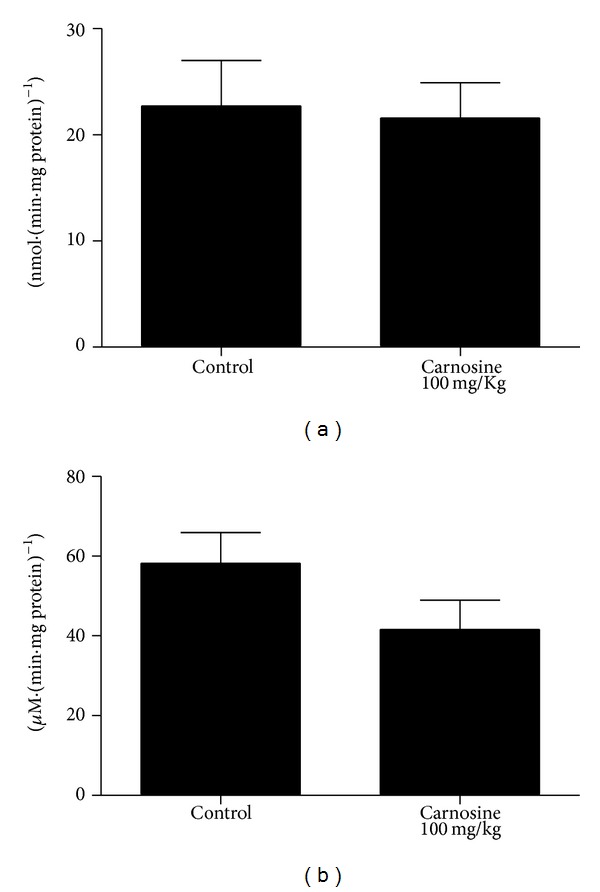
Effect of acute carnosine administration on citric acid cycle enzymes succinate dehydrogenase (a) and malate dehydrogenase (b) activities in skeletal muscle of young rats. Data are mean ± S.E.M. for six independent animals performed in duplicate and are expressed in nmol min^−1^ mg protein^−1^. No significant differences between groups were detected (Student's *t*-test).

**Figure 3:**
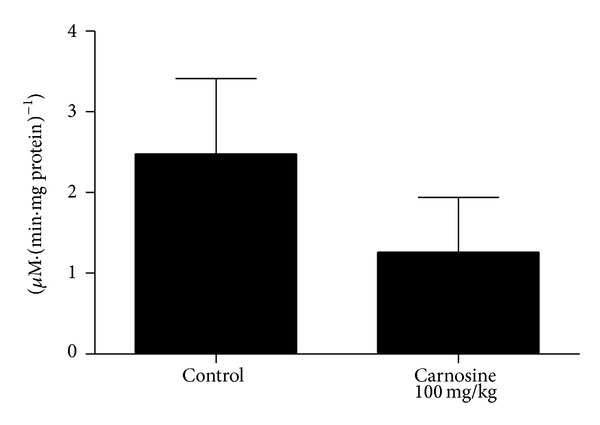
Effect of acute carnosine administration on creatine kinase activity in skeletal muscle of young rats. Data are mean ± S.E.M. for six independent animals performed in duplicate and are expressed in *μ*mol creatine min^−1^ mg protein^−1^. No significant difference between groups was observed (Student's *t*-test).

Initially, we tested the influence of acute carnosine administration. In such condition, we observed a statistically significant decrease of complexes I–III [*t*_(10)_ = 2.41; *P* < 0.05] (Figure 1(a)) and II [*t*_(8)_ = 3.86; *P* < 0.01] (Figure 1(b)) activities in treated animals, as compared to control group rats. Furthermore, the complexes II-III [*t*_(10)_ = 0.918; *P* > 0.05] (Figure 1(c)), succinate dehydrogenase [*t*_(10)_ = 0.211; *P* > 0.05] (Figure 2(a)), malate dehydrogenase [*t*_(10)_ = 1.56; *P* > 0.05] (Figure 2(b)), and creatine kinase [*t*_(10)_ = 1.05; *P* > 0.05] (Figure 3) activities were not significantly different between groups. However, we identified a nonsignificant decrease in complexes II-III and malate dehydrogenase activities in animals that received carnosine, as compared to control rats (Figures 1(c) and 2(b), resp.).

### 3.2. Chronic Carnosine Administration Does Not Affect Energy Metabolism in Rat Skeletal Muscle

**Figure 4:**
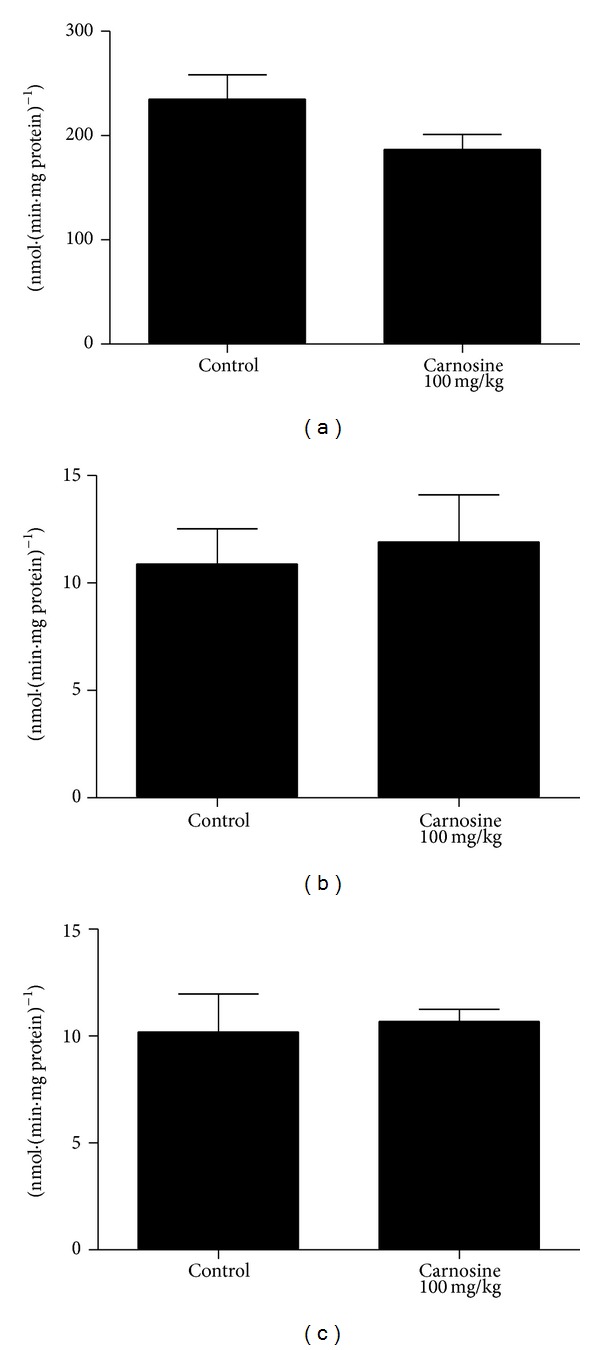
Effect of chronic carnosine administration on respiratory chain complexes I–III (a), II (b), and II-III (c) activities in skeletal muscle of young rats. Data are mean ± S.E.M. for five to six independent animals performed in duplicate and are expressed in nmol min^−1^ mg protein^−1^. No significant difference between groups was detected (Student's *t*-test).

**Figure 5:**
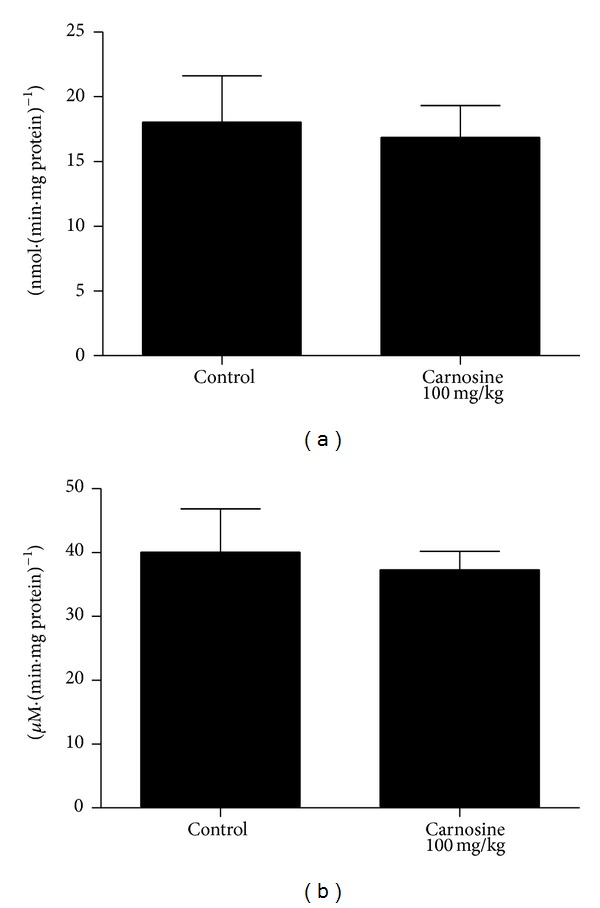
Effect of chronic carnosine administration on citric acid cycle enzymes succinate dehydrogenase (a) and malate dehydrogenase (b) activities in skeletal muscle of young rats. Data are mean ± S.E.M. for six independent animals performed in duplicate and are expressed in nmol min^−1^ mg protein^−1^. No significant difference between groups was observed (Student's *t*-test).

**Figure 6:**
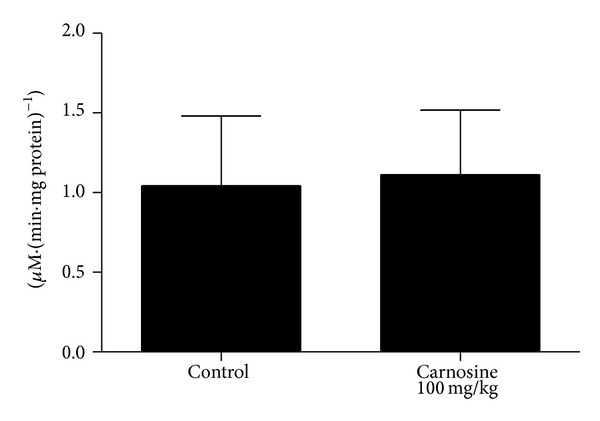
Effect of chronic carnosine administration on creatine kinase activity in skeletal muscle of young rats. Data are mean ± S.E.M. for six independent animals performed in duplicate and are expressed in *μ*mol creatine min^−1^ mg protein^−1^. No significant difference between groups was detected (Student's *t*-test).

The biochemical parameters following chronic carnosine administration were also evaluated. We detected no statistically significant difference in complexes I–III [*t*_(9)_ = −0.169; *P* > 0.05] (Figure 4(a)), II [*t*_(9)_ = −0.383; *P* > 0.05] (Figure 4(b)), II-III [*t*_(9)_ = −0.289; *P* > 0.05] (Figure 4(c)), succinate dehydrogenase [*t*_(10)_ = 0.274; *P* > 0.05] (Figure 5(a)), malate dehydrogenase [*t*_(10)_ = 0.374; *P* > 0.05] (Figure 5(b)), and creatine kinase [*t*_(10)_ = −0.113; *P* > 0.05] (Figure 6) activities between groups in rat skeletal muscle. However, it was observed that there is a trend of decrease of complexes I–III activities in animals receiving carnosine, as compared to control rats (Figure 4(a)).

### 3.3. Mitochondrial Biogenesis

**Figure 7:**
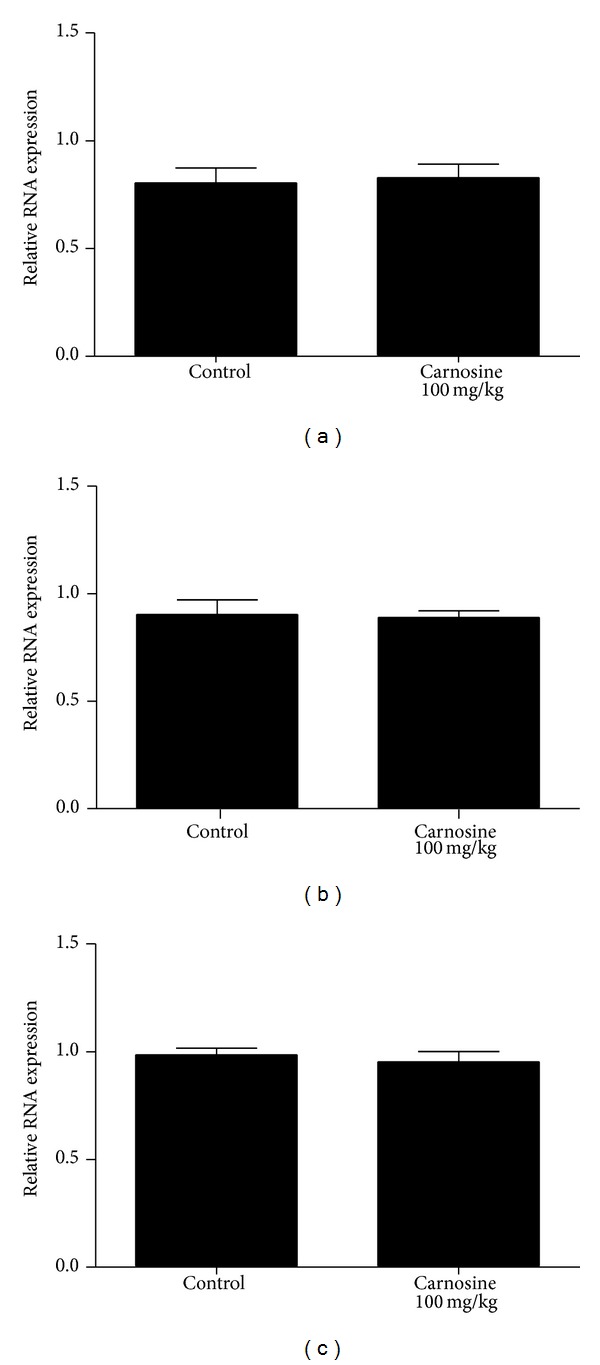
Effect of acute carnosine administration on relative mRNA expression of* NRF-1* (a),* PGC-1*α** (b), and* TFAM* (c) activities in skeletal muscle of young rats. Results are mean ± S.E.M. for four to six independent animals performed in quadruplicate. No significant difference between groups was detected.

**Figure 8:**
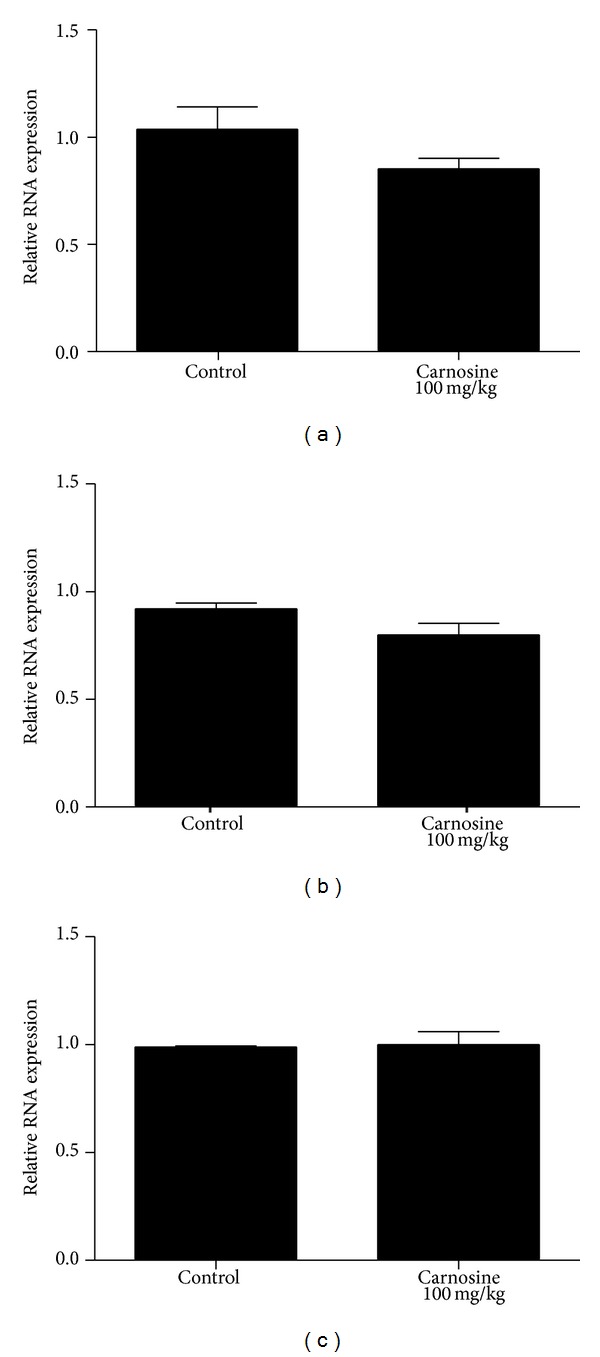
Effect of chronic carnosine administration on relative mRNA expression of* NRF-1* (a),* PGC-1*α** (b), and* TFAM* (c) activities in skeletal muscle of young rats. Results are mean ± S.E.M. for four to six independent animals performed in quadruplicate. No significant difference between groups was detected.

Transcript abundance of key factors involved in mitochondrial biogenesis was evaluated by RT-qPCR in skeletal muscle of animals receiving carnosine acutely (Figure 7) or chronically (Figure 8). As compared to control groups, mRNA levels of* NRF-1*,* PGC-1*α*,* and* TFAM* in the animals receiving carnosine under either regimen of administration were unchanged.

## 4. Discussion

In skeletal muscle tissue of most vertebrates and some invertebrates, the imidazole dipeptide carnosine is synthesized from histidine and *β*-alanine by the ATP-dependent enzyme carnosine synthetase [20]. The biological roles of carnosine and related dipeptides in skeletal muscle remain uncertain, although it is possible that they act as proton buffering agents [4]. Furthermore, there are a growing number of evidences related to carnosine effects on metabolism. Aside from its antioxidant activity [21, 22], carbonyl-scavenger ability has been assigned to carnosine [23] and other imidazole dipeptides [24]. Many of carbonyl compounds are metabolic toxic by-products and, in the bulk of tissues, they are detoxified by oxidoreductases. These enzymes catalyze the oxidation or reduction of aldehydes, or its conjugation with low molecular weight amines and thiols, such as reduced glutathione and imidazole dipeptides [25]. Carnosine was also shown to react with methylglyoxal, a toxic metabolic by-product of glycolysis, as well as to inhibit cancer and aging mechanisms [26] and the cellular respiration regulatory complex mTOR [27], and activate the gluconeogenic enzyme, fructose-1,6-bisphosphatase [28].

It was also shown that carnosine intake significantly diminishes the activity and mRNA expression of malic enzyme, fatty acid synthase, 3-hydroxy-3-methylglutaryl coenzyme A (HMG-CoA) reductase, sterol regulatory element-binding proteins (SREBP-1c), and SREBP-2 in mice that consumed high saturated fat diet [29]. Carnosine was able to inhibit Zn^2+^-induced death of GT1-7 neurons through the inhibition of* GADD34*,* p8* (endoplasmic reticulum stress-related genes), and* Arc* (calcium-related gene) expression [30]. Finally, Asperger and colleagues [31], through a proteomics study with glioblastoma cells receiving carnosine, detected 31 proteins expressed differentially under the influence of dipeptide, including BCL2-associated athanogene 2 and von Hippel-Lindau binding protein 1.

In the present study, we observed a statistically significant decrease of complexes I–III and II activities in animals receiving carnosine acutely, compared to control group rats. Additionally, we detected no significant difference in complexes II-III, citric acid cycle enzymes, and creatine kinase activities between groups. Finally, we were also not able to find any significant alteration of respiratory chain complexes, citric acid cycle enzymes, and creatine kinase activities. Since several evidences suggest that many of the physiological effects of carnosine administration initiate at transcriptional level, as shown by changes in mRNA levels of HIF-1*α* [32], runt-related transcription factor-2/core binding factor alpha-1 (RUNX2/Cbf*α*1),* Sox9* [33], Hsp70, and* SOD2* [34], mitochondrial biogenesis could explain these findings. Nevertheless, it was found that mRNA levels of* NRF-1*,* PGC-1*α*,* and* TFAM* in skeletal muscle of rats receiving carnosine acutely or chronically were similar to those measured in control animals.

Our finding showing that the malate dehydrogenase activity presented no statistically significant difference between groups in both treatments corroborates, at least in part, a literature report. It was found that a patient with serum carnosinase deficiency exhibited malate dehydrogenase and lactate dehydrogenase activities within normal limits in liver, kidney, and spleen specimens, despite the fact that patients with this disorder present high levels of carnosine in their plasma (carnosinemia) [8].

We also observed that succinate dehydrogenase activity in skeletal muscle of carnosine group was not significantly different from those in control group, in animals subjected to either acute or chronic carnosine administration. On the other hand, a literature report showed that rats chronically intoxicated with ethanol presented reduced activity of some hepatic enzymes, including succinate dehydrogenase [35]. It was also showed in the same study that carnosine load for two weeks previously or simultaneously with ethanol intoxication prevents or reverses the toxicity of such alcohol on this enzyme activity.

It should be emphasized that, in skeletal muscle of rats receiving carnosine acutely, a statistically significant decrease in complexes I–III and II activities was identified, as well as a trend of decrease in complexes II-III activities, relatively to control group. However, rats receiving carnosine chronically showed only a trend of decrease in complexes I–III activities in skeletal muscle, as compared to control group, whereas the other complexes activities were not different between groups.

The same pattern of alteration was also observed in malate dehydrogenase activity, which was found increased in rats receiving carnosine acutely and unaltered in animals receiving the dipeptide chronically. Thus, it may be proposed that skeletal muscle is able to adapt to high carnosine levels, recovering the impaired enzyme activities back to control conditions. A putative mechanism involved in such adaptation is increased mitochondrial synthesis, since the enzymes affected by carnosine administration present mitochondrial localization. Considering that carnosine administration increases vimentin synthesis [36] and that vimentin is a cytoskeleton protein that may be related to mitochondrial motility and localization [37], it is possible that this process is mediated by increased vimentin synthesis. However, data on* NRF-1*,* PGC-1*α**, and* TFAM* mRNA levels suggest that carnosine interferes with these enzymatic activities through mechanisms unrelated to mitochondrial biogenesis, but the influence of carnosine on mitophagy cannot be ruled out.

At present, we cannot ascertain the pathophysiological relevance of our findings. In this communication, we induced carnosinemia in the animals by administering carnosine intraperitoneally. With respect to this fact, it should be mentioned that carnosine dietary administration elicits increase of its content in human skeletal muscle [38]. The carnosine levels in this tissue are situated within 30–50 mmol/kg of wet weight [39] and are prone to alterations according to diet, gender, and age of subject [40]. Additionally, in our study, carnosine is increased in skeletal muscle of rat, such as occurrence in patients with carnosinemia.

## 5. Conclusion

It is here shown that carnosine administration impairs electron transfer through the mitochondrial respiratory chain in skeletal muscle of young rats, without changing mitochondrial biogenesis-related transcription factors. At least in part, this finding does not corroborate some reports, which indicate that this dipeptide increases the efficiency and intensity of oxidative phosphorylation in rats adapted to hypobaric hypoxia [41]. The human carnosinase levels are almost undetectable in plasma of neonates, increasing according to aging [6], rendering young individuals less prone to metabolize carnosine. In case these findings are confirmed by further studies and ATP depletion is also observed, it is possible that an energy dysfunction, secondary to carnosine accumulation, may aid to explain some of muscle symptoms commonly observed in young patients with serum carnosinase deficiency.

## Figures and Tables

**Table 1:** Primer sequences for RT-qPCR experiments included in the study.

Gene	Forward primer	Reverse primer
*Gapd* ^ a^	5′-GCTAAGCAGTTGGTGGTGCA-3′	5′-TCACCACCATGGAGAAGGC-3′
*Hprt1* ^ a^	5′-GCAGACTTTGCTTTCCTTGG-3′	5′-GCAGACTTTGCTTTCCTTGG-3′
*NRF-1* ^ b^	5′-TTACTCTGCTGTGGCTGATGG-3′	5′-CCTCTGATGCTTGCGTCGTCT-3′
*PGC-1*α** ^ c^	5′-CGTTACACCTGTGACGCTTTCGCTG-3′	5′-CATACTTGCTCTTGGTGGAAGCAGG-3′
*TFAM* ^ c^	5′-AATTGAAGCTTGTAAATCAGGCTTGG-3′	5′-CGGATGAGATCACTTCGCCCAAC-3′

According to ^a^Bonefeld et al., 2008 [12]; ^b^Zhang et al., 2012 [13]; ^c^designed by authors.
